# Exit of pediatric pre-B acute lymphoblastic leukaemia cells from the bone marrow to the peripheral blood is not associated with cell maturation or alterations in gene expression

**DOI:** 10.1186/1476-4598-7-67

**Published:** 2008-08-11

**Authors:** Frida Hansson, Jacek Toporski, Robert Månsson, Bertil Johansson, Ulrika Norén-Nyström, Sten Eirik W Jacobsen, Thomas Wiebe, Marcus Larsson, Mikael Sigvardsson, Anders Castor

**Affiliations:** 1 Department of Stem Cell Biology, Lund Center for Stem Cell Biology and Cell Therapy, Lund University, Lund, Sweden; 2 Department of Pediatrics, Section of Pediatric Oncology/Hematology, Lund University Hospital, Lund, Sweden; 3 Department of Clinical Genetics, Lund University Hospital, Lund, Sweden; 4 Department of Clinical Sciences, Pediatrics, University of Umeå, Umeå, Sweden; 5 University of Health Sciences, Linköping University, Sweden

## Abstract

**Background:**

Childhood pre-B acute lymphoblastic leukemia (ALL) is a bone marrow (BM) derived disease, which often disseminates out of the BM cavity, where malignant cells to a variable degree can be found circulating in the peripheral blood (PB). Normal pre-B cells are absolutely dependent on BM stroma for survival and differentiation. It is not known whether transformed pre-B ALL cells retain any of this dependence, which possibly could impact on drug sensitivity or MRD measurements.

**Results:**

Pre-B ALL cells, highly purified by a novel method using surface expression of CD19 and immunoglobulin light chains, from BM and PB show a very high degree of similarity in gene expression patterns, with differential expression of vascular endothelial growth factor (VEGF) as a notable exception. In addition, the cell sorting procedure revealed that in 2 out of five investigated patients, a significant fraction of the malignant cells had matured beyond the pre-B cell stage.

**Conclusion:**

The transition of ALL cells from the BM into the circulation does not demand, or result in, major changes of gene expression pattern. This might indicate an independence of BM stroma on the part of transformed pre-B cells, which contrasts with that of their normal counterparts.

## Background

Children suffering from acute lymphoblastic leukemia, in the recent past an inevitably fatal disease, have experienced a dramatically improved outcome during the past 4 decades, such that four out of five newly diagnosed pediatric patients today can expect to be cured [1-6]. However, in order to further improve the prognosis for children with ALL, it is crucial to learn more about the molecular consequences and causes of malignant transformation. In addition to leading to an uncontrolled cell growth of pre-B ALL cells, transformation also results in a pronounced block of cell differentiation. This developmental disturbance is also reflected in the primary anatomical location of the leukemic cells being the bone marrow (BM), which also is the primary site for normal progenitor B-lymphocytes. Hence, it is reasonable to assume that the transformed cells in general maintain several of the features of the B-cell progenitors and thus utilize the presence of growth factors in the BM in a fashion similar to a normal cell. However, even though the BM is the primary site for leukemic cells, extramedullary locations, including peripheral blood (PB), often contains cells related to the malignant clone in the BM. Given the requirement of stroma signalling for normal pre-B cells, it is not obvious that ALL cells residing in the BM are similar to ALL cells in the circulation. Malignant cells in these two locations could differ with regard to differentiation stage, cell cycle status or proneness to apoptosis, which might influence drug sensitivity and thus also minimal residual disease (MRD) measurements.

In order to establish the relationship between ALL cells in the BM and in the PB, and to resolve how the anatomical location is reflected in the overall gene expression pattern of a pre-B ALL cell, we developed a purification approach based on the presumption that the transformed cells express the lineage marker CD19, but due to the developmental block lack the expression of Immunoglobulin light chain (IgL) protein, normally not expressed until later stages of development [7], on the cell surface. This allowed us to purify leukemic cells from both BM and PB in the same patients, and subsequent gene expression analysis revealed that the overall gene expression pattern in transformed cells in PB overlaps with that of phenotypically similar cells in the BM. These data suggest the ability of leukemic blasts to migrate freely independently of any putative niche otherwise restricting normal pre-B cells to the BM.

## Patients and methods

### Patients

BM and PB were obtained at diagnosis and at remission from five children with ALL, and three children diagnosed with non-malignant disease, after informed consent and with the approval of the research ethics committee at Lund University. Patients were selected based on availability of enough cells after diagnostic work-up, and on the presence of a chromosomal aberration, which could be detected by FISH.

### Cell separation, phenotyping, and sorting

BM and PB mononuclear cells were isolated, frozen/thawed and stained as previously described [8]. Cells were stained with anti-CD19-allophycocyanin (APC), anti-κ-fluorescein isothiocyanate (FITC) and anti-λ-phycoerythrin (PE), all from Becton Dickinson (BD). Dead cells were excluded by staining with 7-aminoactinomycin D (7-AAD, Sigma). Cells were sorted on a FACS DiVa cell sorter (BD), and data analysis was done with the Cell Quest (BD) software.

### FISH analyses

Interphase FISH analyses were performed as previously described[8], using commercially available probes (Vysis) for the respective genetic abnormalities, i.e., *ETV6 *for dic(7;12)(p11;p11) (resulting in loss of the *ETV6 *gene), *ETV6/RUNX1 *for t(12;21)(p13;q22), *TCF3 *for t(1;19)(q23;p13), and a chromosome 21 probe for high hyperdiploidy (> 50 chromosomes. 200–300 nuclei were analyzed in each sample.

### Microarray

RNA was extracted as previously described [9], labeled and amplified according to Affymetrix™; Small Sample Labeling Protocol v.2. Affymetrix HG-U133 plus 2.0 Chips were normalized using invariant set normalization and probe level expression values were calculated using the PM-MM model provided by the dCHIP software [10].

### Q-PCR

RNA was isolated by sorting of cells into RLT buffer for subsequent RNA purification using the RNAeasy kit (Qiagen Inc) according to the manufacturers instructions for RNA purification from 10 000 cells. cDNA was generated by annealing total RNA from 10 000 cells to 0,5 μg of random hexamers in 10 μl DEPC-treated water. Reverse transcriptase reactions were performed with 200 units of SuperScript Reverse Transcriptase (Life Technologies) in the manufacturers' buffer supplemented with 0,5 mM dNTP, 10 mM DTT and 20 units RNase inhibitor (Boeringer Mannheim, Bromma, Sweden) in a total volume of 20 μl. The real-time polymerase chain reaction (PCR) was based on the Taqman™ technology (Applied Biosystems, Stockholm, Sweden). The threshold cycles (Ct) for the endogenous control HPRT mRNA and the target signals were determined and the relative RNA quantification was calculated using the comparative Ct method as 2^-ΔCt ^where ΔCt is Ct (target)-Ct (HPRT).

Oligonucleotides for quantitative Taqman™ real time PCR were ordered as Assay on demand (Applied Biosystems).

TaqMan^® ^probes used were: HPRT1 (Hs99999909_m1) and VEGF (Hs00900055_m1).

## Results and discussion

### Cell sorting based on Ig-light chain expression on B-lineage cells allows for the purification of pre-B ALL cells

**Figure 1:**
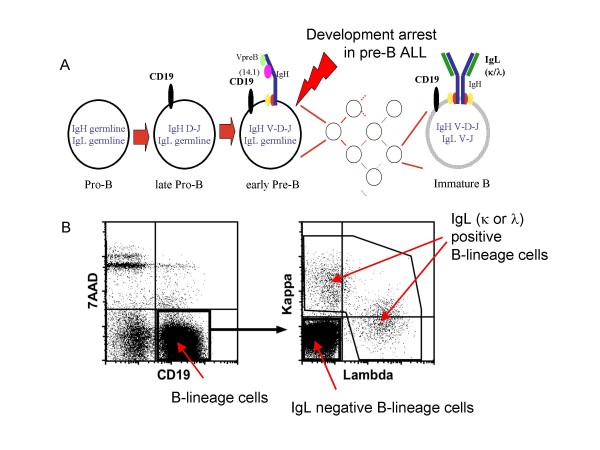
**Sorting of pre-B and immature B cells**. Panel **(A) **display a schematic drawing of the B-lymphoid developmental pathway. **(B) **Representative FACS plot of BM MNCs from a pediatric patient diagnosed with a pre-B ALL, stained with antibodies against CD19 and IgL (κ and λ). Gates for sorting are depicted.

In order to perform a valid comparison of gene expression patterns in BM-derived leukemic cells to those derived from PB, we needed to establish a protocol that would allow us to identify and purify leukemic cells in BM and PB. We also wanted to sort a comparable cell population from BM of the same patients in remission, both in order to identify tumor-specific features and to obtain an impression of the effect that the genetic background might have on the gene expression patterns in the normal regenerating BM. In order to achieve this we used the fact that IgL typically is not rearranged, and thus not expressed on the cell surface, until the immature B-cell stage [7] (Figure 1A), and since pre-B ALL cells are blocked in their differentiation prior to that stage they would not be expected to display any IgL on the cell surface. Hence, we sorted mononuclear cell preparations from BM and PB from five patients based on positive expression of the B-lineage marker CD19 and lack of expression of surface IgL (Figure 1B). The percentage of CD19 positive cells varied from 63 to 91 percent in BM (Table 1), and from 9.2 to 82% in PB (Table 1). A minor part (2–15%) of the BM CD19^+ ^cells also expressed IgL chains (Table 1) while the percentage of IgL positive cells varied from 5 to 90% in the PB. These data show that the composition of cells in BM and PB at the time of diagnosis is highly variable, and thus highlight the need for purification of cells before any relevant comparisons of function or gene expression patterns can be made. All the investigated samples carried chromosomal aberrations which allowed us to analyze the clonal involvement of the sorted cell populations at a single cell level by FISH. This revealed that while the percentage of cells carrying a chromosomal abnormality in the IgL positive fractions varied from 1.5 to 33%, the FISH positive fraction of the IgL negative cells was between 96 and 100%. When comparing BM and PB cells from individual patients, it is of note that the surface expression pattern of CD19 and IgL characteristic of pre-B cells is retained in extramedullary circulating leukemic blasts. Interestingly, in some of the patients the leukemic clone contained a significant fraction of cells expressing an IgL, indicating developmental progression into the B cell stage, and suggesting that the developmental block is incomplete.

**Table 1:** Clonal involvement of sorted IgL^+ ^and IgL^- ^cells.

**Patient**	**Translocation**	**%CD19^+ ^cells** ** of all BM MNCs**	**%IgL^+ ^cells of** ** all CD19^+ ^cells**	**Population**	**% FISH**^+^
A	dic(7;12)(p11;p11)	76	15	CD19^+^IgL^-^	99
				CD19^+^IgL^+^	1,9
B	t(12;21)(p13;q22)	63	12	CD19^+^IgL^-^	99
				CD19^+^IgL^+^	2,7
C	t(1;19)(q23;p13)	84	7	CD19^+^IgL^-^	98
				CD19^+^IgL^+^	27
D	High hyperdiploidy	87	7	CD19^+^IgL^-^	99
				CD19^+^IgL^+^	25
E	t(12;21)(p13;q22)	91	2	CD19^+^IgL^-^	100
				CD19^+^IgL^+^	3

**Patient**	**WBC ×10^9^/l**	**%CD19^+ ^cells ** **of all PB MNCs**	**%IgL^+ ^cells of** ** all CD19^+ ^cells**	**Population**	**% FISH**^+^

A	3.5	54	52	CD19^+^IgL^-^	97
				CD19^+^IgL^+^	4,2
B	2.1	9,2	90	CD19^+^IgL^-^	ND
				CD19^+^IgL^+^	ND
C	70	57	20	CD19^+^IgL^-^	96
				CD19^+^IgL^+^	33
D	23	81	12	CD19^+^IgL^-^	99
				CD19^+^IgL^+^	17
E	29	82	5	CD19^+^IgL^-^	100
				CD19^+^IgL^+^	8

MNCs from five patients (A-E) analyzed for phenotype by FACS and clonal involvement in IgL^+ ^and IgL^- ^cells by detection of the corresponding chromosome abnormality by FISH. Upper panel shows data from BM at diagnosis and lower panel from PB at diagnosis.

### ALL cells from BM and PB display a common but patient specific transcriptome

**Figure 2:**
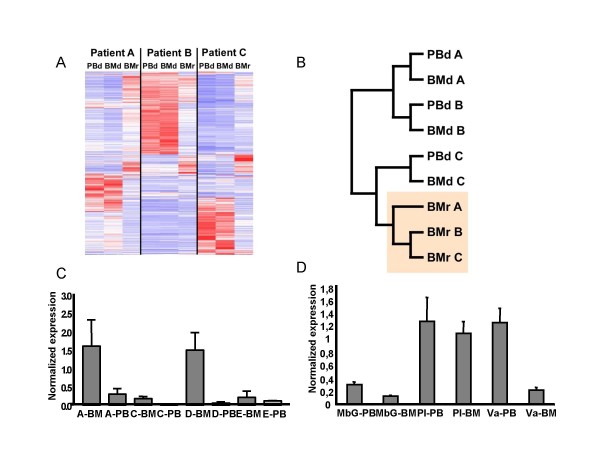
**Microarray analysis of patient samples**. **(A) **Hierarchical clustering of gene expression data obtained from purified diagnostic blood (PBd) and bone marrow (BMd) as well as remission BM (BMr) cells from three investigated patients A-C. (Genes used for clustering satisfied the following two criterions; > 0.5 SD/MEAN across samples, and ≥ 100 expression units in ≥ 10% of arrays used.) **(B) **Overall hierarchical relationship in gene expression patterns between the samples. **(C) **Q-PCR analysis of normalized expression levels of VEGF in BMd and PBd in 4 patients (A, C-E). The data presented is triplicates from one representative out of two experiments. Error bars indicate standard deviation. (**D**) Q-PCR analysis of normalized expression levels of VEGF in BM and PB in 3 patients diagnosed with other disease than ALL. Mb Gauchers (MbG), Post Infection (PI) or Vasculitis (VA) as indicated. The data presented is triplicates and error bars indicate standard deviation.

Having established a protocol with which tumor cells can be purified from both BM and PB to almost homogeneity, we next performed gene expression micro-array analysis, using Affymetrix, to compare the global gene expression pattern between leukemic cells harvested from BM and PB in three of the patients (Figure 2A and 2B). In addition, we sorted CD19^+^IgL^- ^BM cells for micro array analysis from the same three patients when in clinical and molecular (RT-PCR) remission for use as a normal reference. Analysis of the data revealed highly related expression patterns in cells obtained from all three patients in remission, suggesting that the impact of patient specific genetic variation on gene expression patterns in these cells is low (Figure 2A and 2B). In contrast, the expression profiles between patient samples at diagnosis showed significant differences, in line with observed differences between subgroups of ALL previously published [11-13]. Within the same patient, however, the expression patterns between BM and PB cells were almost identical (Figure 2A and 2B), suggesting that the different location of the cells do not involve any major changes in the transcriptome, and further supports the idea that exit from the bone marrow environment does not involve major changes of the cellular phenotype.

### VEGF is differentially expressed in BM as compared to PB pre-B ALL cells

In order to identify genes with differential expression patterns associated with the anatomical location, we searched the data set for genes with an expression difference greater than a factor two on comparison between diagnostic BM and PB across all three patients. The single transcript fulfilling these criteria was coding for vascular endothelial growth factor (VEGF), with an elevated expression in BM as compared to PB. In order to verify and extend this finding, we analysed the expression of VEGF by Q-PCR analysis of RNA from purified leukemic BM and PB cells from patient A, C, D and E (Figure 2C) (Patient B could not be analyzed due to lack of material). These experiments verified that the expression level of VEGF was reduced in PB as compared to BM in all the patients. The relative levels of VEGF transcripts in the BM were, however, largely different between patients, arguing against a general super activation of VEGF in pre-B ALL BM cells. In order to investigate if this differential expression pattern is a common phenomena or something related to ALL, we analysed the expression of VEGF in CD19+IgL- cells in the BM and PB from children with other diagnoses (vasculitis, Mb Gaucher and post infectious peripheral neutropenia) (Figure 2D). In all three cases VEGF expression was comparable or higher in the PB than in the BM. This indicates that the higher level of VEGF expression in BM is restricted to ALL. An elevated VEGF expression in BM as compared to PB is an intriguing finding in light of recent observations of an autocrine survival loop that involves VEGF in normal HSCs [14] as well as in acute leukemias [15], and with an increased production of B-cells upon continuous VEGF stimulation [16]. The difference in expression level could indicate a segregation of the leukemic blasts into mitotically more active cells residing in the BM, or it could be an effect caused by relative hypoxia in the ALL BM, which induces VEGF transcription through hypoxia-inducible factor 1 (HIF-1) [17].

## Conclusion

We here report a protocol for purification of ALL cells from different anatomical localisations, which we in the present work has used to compare gene expression patterns in leukemic blasts harvested from BM and PB. This comparison revealed that transition of ALL cells from the bone marrow to the circulation is not a result of, or contributes to, progressed differentiation, but rather suggests an inherent ability of leukemic blasts to exist in a microenvironment not supportive for normal cells at a corresponding maturational stage. Further, the sorting of CD19+ lymphoid cells at different maturational stages, as defined by the presence or absence of IgL, followed by single cell analysis of leukaemia specific chromosomal aberrations by FISH, interestingly revealed that both blood and BM from two of the patients (C and D) contained a significant fraction of clonal IgL^+ ^cells. This indicates that ALL cells may not always be as stringently blocked in development as commonly thought, but may retain some ability to progress beyond the pre-B cell stage into more mature stages of development.

## Competing interests

The authors declare that they have no competing interests.

## Authors' contributions

FH participated in the design of the study, and performed and analyzed the PCR and microarray studies. JT performed the cell sorting and FACS analysis. RM performed and analyzed the PCR and microarray studies. BJ performed and analyzed the FISH studies. UN-N has contributed study material, and has been involved in revising the manuscript. SEJW contributed during study design. TW contributed study material, and was involved in study conception. ML participated in study conception, design and coordination. MS was involved in study conception and design, analyzed data and critically revised the manuscript. AC performed cell sorting and FACS analysis, participated in study design and coordination, analyzed data and drafted the manuscript. All authors read and approved the final manuscript.

## References

[B1] Schrappe M, Reiter A, Zimmermann M, Harbott J, Ludwig WD, Henze G, Gadner H, Odenwald E, Riehm H (2000). Long-term results of four consecutive trials in childhood ALL performed by the ALL-BFM study group from 1981 to 1995. Berlin-Frankfurt-Munster. Leukemia.

[B2] Gaynon PS, Trigg ME, Heerema NA, Sensel MG, Sather HN, Hammond GD, Bleyer WA (2000). Children's Cancer Group trials in childhood acute lymphoblastic leukemia: 1983-1995. Leukemia.

[B3] Silverman LB, Declerck L, Gelber RD, Dalton VK, Asselin BL, Barr RD, Clavell LA, Hurwitz CA, Moghrabi A, Samson Y, Schorin MA, Lipton JM, Cohen HJ, Sallan SE (2000). Results of Dana-Farber Cancer Institute Consortium protocols for children with newly diagnosed acute lymphoblastic leukemia (1981-1995). Leukemia.

[B4] Gustafsson G, Schmiegelow K, Forestier E, Clausen N, Glomstein A, Jonmundsson G, Mellander L, Makipernaa A, Nygaard R, Saarinen-Pihkala UM (2000). Improving outcome through two decades in childhood ALL in the Nordic countries: the impact of high-dose methotrexate in the reduction of CNS irradiation. Nordic Society of Pediatric Haematology and Oncology (NOPHO). Leukemia.

[B5] Pui CH, Sandlund JT, Pei D, Campana D, Rivera GK, Ribeiro RC, Rubnitz JE, Razzouk BI, Howard SC, Hudson MM, Cheng C, Kun LE, Raimondi SC, Behm FG, Downing JR, Relling MV, Evans WE (2004). Improved outcome for children with acute lymphoblastic leukemia: results of Total Therapy Study XIIIB at St Jude Children's Research Hospital. Blood.

[B6] Pui CH, Relling MV, Downing JR (2004). Acute Lymphoblastic Leukemia. N Engl J Med.

[B7] Ghia P, ten Boekel E, Sanz E, de la Hera A, Rolink A, Melchers F (1996). Ordering of Human Bone Marrow B Lymphocyte Precursors by Single-Cell Polymerase Chain Reaction Analyses of the Rearrangement Status of the Immunoglobulin H and L Chain Gene Loci. J Exp Med.

[B8] Castor A, Nilsson L, Astrand-Grundstrom I, Buitenhuis M, Ramirez C, Anderson K, Strombeck B, Garwicz S, Bekassy AN, Schmiegelow K, Lausen B, Hokland P, Lehmann S, Juliusson G, Johansson B, Jacobsen SE (2005). Distinct patterns of hematopoietic stem cell involvement in acute lymphoblastic leukemia. Nat Med.

[B9] Adolfsson J, Mansson R, Buza-Vidas N, Hultquist A, Liuba K, Jensen CT, Bryder D, Yang L, Borge OJ, Thoren LAM (2005). Identification of Flt3+ Lympho-Myeloid Stem Cells Lacking Erythro-Megakaryocytic Potential:  A Revised Road Map for Adult Blood Lineage Commitment. Cell.

[B10] Li C, Wong WH (2001). Model-based analysis of oligonucleotide arrays: expression index computation and outlier detection. Proc Natl Acad Sci U S A.

[B11] Yeoh EJ, Ross ME, Shurtleff SA, Williams WK, Patel D, Mahfouz R, Behm FG, Raimondi SC, Relling MV, Patel A, Cheng C, Campana D, Wilkins D, Zhou X, Li J, Liu H, Pui CH, Evans WE, Naeve C, Wong L, Downing JR (2002). Classification, subtype discovery, and prediction of outcome in pediatric acute lymphoblastic leukemia by gene expression profiling. Cancer Cell.

[B12] Ross ME, Zhou X, Song G, Shurtleff SA, Girtman K, Williams WK, Liu HC, Mahfouz R, Raimondi SC, Lenny N, Patel A, Downing JR (2003). Classification of pediatric acute lymphoblastic leukemia by gene expression profiling. Blood.

[B13] Andersson A, Olofsson T, Lindgren D, Nilsson B, Ritz C, Eden P, Lassen C, Rade J, Fontes M, Morse H, Heldrup J, Behrendtz M, Mitelman F, Hoglund M, Johansson B, Fioretos T (2005). Molecular signatures in childhood acute leukemia and their correlations to expression patterns in normal hematopoietic subpopulations. Proc Natl Acad Sci.

[B14] Gerber HP, Malik AK, Solar GP, Sherman D, Liang XH, Meng G, Hong K, Marsters JC, Ferrara N (2002). VEGF regulates haematopoietic stem cell survival by an internal autocrine loop mechanism. Nature.

[B15] Dias S, Hattori K, Zhu Z, Heissig B, Choy M, Lane W, Wu Y, Chadburn A, Hyjek E, Gill M, Hicklin DJ, Witte L, Moore MA, Rafii S (2000). Autocrine stimulation of VEGFR-2 activates human leukemic cell growth and migration. J Clin Invest.

[B16] Gabrilovich D, Ishida T, Oyama T, Ran S, Kravtsov V, Nadaf S, Carbone DP (1998). Vascular endothelial growth factor inhibits the development of dendritic cells and dramatically affects the differentiation of multiple hematopoietic lineages in vivo. Blood.

[B17] Semenza GL (2000). HIF-1 and human disease: one highly involved factor. Genes Dev.

